# Comparative Oncology: Evaluation of 2-Deoxy-2-[18F]fluoro-D-glucose (FDG) Positron Emission Tomography/Computed Tomography (PET/CT) for the Staging of Dogs with Malignant Tumors

**DOI:** 10.1371/journal.pone.0127800

**Published:** 2015-06-12

**Authors:** Stefanie M. F. Seiler, Christine Baumgartner, Johannes Hirschberger, Ambros J. Beer, Andreas Brühschwein, Nina Kreutzmann, Silja Laberke, Melanie C. Wergin, Andrea Meyer-Lindenberg, Johanna Brandl, Anne-Kathrin von Thaden, Eliane Farrell, Markus Schwaiger

**Affiliations:** 1 Clinic of Small Animal Medicine, Center for Clinical Veterinary Medicine, Ludwig, Maximilians Universität, 80539, Munich, Germany; 2 Center of Preclinical Research, Technische Universität München, 81675, Munich, Germany; 3 Clinic for Small Animal Surgery and Reproduction, Veterinary Faculty, Ludwig, Maximilians Universität, 80539, Munich, Germany; 4 Department of Nuclear Medicine, Technische Universität München, 81675, Munich, Germany; Stanford University School of Medicine, UNITED STATES

## Abstract

**Introduction:**

2-Deoxy-2-[18F]fluoro-D-glucose PET/CT is a well-established imaging method for staging, restaging and therapy-control in human medicine. In veterinary medicine, this imaging method could prove to be an attractive and innovative alternative to conventional imaging in order to improve staging and restaging. The aim of this study was both to evaluate the effectiveness of this image-guided method in canine patients with spontaneously occurring cancer as well as to illustrate the dog as a well-suited animal model for comparative oncology.

**Methods:**

Ten dogs with various malignant tumors were included in the study and underwent a whole body FDG PET/CT. One patient has a second PET-CT 5 months after the first study. Patients were diagnosed with histiocytic sarcoma (n = 1), malignant lymphoma (n = 2), mammary carcinoma (n = 4), sertoli cell tumor (n = 1), gastrointestinal stromal tumor (GIST) (n = 1) and lung tumor (n = 1). PET/CT data were analyzed with the help of a 5-point scale in consideration of the patients’ medical histories.

**Results:**

In seven of the ten dogs, the treatment protocol and prognosis were significantly changed due to the results of FDG PET/CT. In the patients with lymphoma (n = 2) tumor extent could be defined on PET/CT because of increased FDG uptake in multiple lymph nodes. This led to the recommendation for a therapeutic polychemotherapy as a treatment. In one of the dogs with mammary carcinoma (n = 4) and in the patient with the lung tumor (n = 1), surgery was cancelled due to the discovery of multiple metastasis. Consequently no treatment was recommended.

**Conclusion:**

FDG PET/CT offers additional information in canine patients with malignant disease with a potential improvement of staging and restaging. The encouraging data of this clinical study highlights the possibility to further improve innovative diagnostic and staging methods with regard to comparative oncology. In the future, performing PET/CT not only for staging but also in therapy control could offer a significant improvement in the management of dogs with malignant tumors.

## Introduction

Cancer is the leading cause of death in dogs as 27% die from malignant tumors [1]. Therefore one of the main goals in veterinary oncology is improving diagnostic techniques to allow earlier recognition of tumors in dogs and cats which is essential for successful therapeutic interventions.

PET/CT is a new attractive method for non-invasive staging, restaging and monitoring of response to therapy in veterinary medicine. In human medicine, the use of PET/CT was first described in 2000. Since then, whole body PET/CT in humans has shown to be a useful tool for diagnosis, staging and monitoring of various malignancies [2–4]. This technique significantly improves imaging of tumors compared with either PET or CT alone [5,6]. However, the use of PET/CT in veterinary patients has been limited due to the lack of access to dual-modality hybrid PET/CT scanners. The high costs of the equipment and the production of tracers immediately before their use precluded a widespread use of this technique in veterinary medicine up to now.

The use of PET/CT in dogs is reported in a number of studies [7–19]. The first use of FDG-PET in dogs with spontaneously occurring osteosarcoma and lymphoma was described in 1981. This study demonstrated increased FDG uptake in spontaneous canine malignancies. Increased FDG uptake was present in both the primary tumors and in metastatic lesions [11]. Whole body PET/CT was performed in healthy dogs to evaluate the physiologic distribution of FDG in thoracic and abdominal organs, including liver, spleen, adrenal glands and heart muscle [13,18]. These data provide a basis for the interpretation of pathologic changes that can be imaged by using this technique. Increased uptake of FDG could be demonstrated in canine lymphoma [11], canine cutaneous mast cell tumor [9], and in various solid tumors (sarcomas, carcinomas) [15]. In all of these studies, PET seemed to be an efficient and useful tool in canine patients. This non- invasive diagnostic method allowed imaging of physiologic and pathologic processes on a molecular level.

This study was designed by a research cooperation between human and veterinarian institutes with a keen interest in the improvement of diagnostic and therapeutic methods in comparative oncology. The importance of comparative oncology has increased during the last couple of years. Because of genetic and biologic similarities regarding certain tumor types, the dog is particularly well suited as a model for human tumors [20]. Using dogs with spontaneously arising tumors as a model for human tumors offers advantages over the use of transgenic animal models due to the more comparable behavior and biologic properties of the tumors. Therefore, comparative oncology is an important innovative tool that can bridge the gap between basic cancer research and the clinic with benefits for both human and veterinary medicine [21,22]. Results acquired from pet animals with naturally occurring diseases like spontaneous tumors can improve the evaluation of the effectiveness and safety of new cancer therapy options for human and animal patients [23,24].

The aims of this pilot study were to evaluate the feasibility and efficacy of whole body FDG PET/CT for staging and restaging in dogs with various tumor types. Furthermore, this study should illustrate the canine patient with spontaneously occurring caner as an animal model for comparative oncology. In addition the study served to establish a basis for further comparative studies at the participating institutions.

## Material and Methods

### Ethics Statement

All experiments described in this paper were carried out with the approval of, and in accordance with the recommendations laid down by the “Deutsches Tierschutzgesetz”. These guidelines are equivalent to the “Animal Welfare Act”. This study was approved by the Animal Welfare Officer of the Centre of Preclinical Research, Technische Universität München, where the anesthesia and the PET/CT scans of the dogs were performed, in addition to the Government of Upper Bavaria.

### Patient Population

Ten dogs aged 8.75 ± 1.9 y and weighing 25.8 ± 11.3 kg were included in the study. Five of the dogs were male (one intact and four neutered) and five female (three intact and two spayed). Breeds included Flat Coated Retriever (n = 2), Golden Retriever (n = 1), Beauceron (n = 1), Standard Schnauzer (n = 1) and mixed breed dogs (n = 5).

Informed owner’s consent was required for all dogs participating in the study. Exclusion criteria were a concurrent diagnosis of diabetes mellitus, pregnancy, and an anesthetic risk classification of ASA IV or V. The ASA classification serves to divide the patients into different groups according to their physical state and so enables the risk of narcosis to be assessed. Class I-III describes patients ranging from organically healthy, clinically unremarkable to those with marked organic / clinical alterations. Class IV describes patients with severe organic damage and poor vital functions. Class V means that there is an acute risk to life with severe organic damage. The owners of all dogs were advised to avoid vigorous physical activity for their dogs during 24 hours before the scan to prevent an excessive FDG uptake within the muscle tissue.

In the preliminary assessment, all dogs underwent a physical examination and a one-view thoracic radiograph in right lateral recumbency. Blood samples were taken and complete blood counts as well as a plasma biochemistry profile were performed. The blood glucose level in the canine patients before the scan was 4.85 mmol/l ± 1.3. According to the results of these examinations, the dogs’ anesthetic risks were classified using the ASA classification system. Depending on size and localization of the tumor, samples for cytological or histopa thologic evaluation were taken either by fine needle aspiration, by needle core biopsy or by excisional biopsy.

The patient with the histiocytic sarcoma was the only patient who underwent a PET/CT examination twice. The reason for this was a restaging or monitoring of the effects of medication. The prognosis for a histiocytic sarcoma is very poor with a very high risk of metastasization and relapse. The median survival time is approximately 6 months [25]. There were three months between the first and second scan and already 5 months since the diagnosis was made. This is why we believed that a rescan was indicated as improved staging and as a progress check using PET/CT.

### Anesthesia Protocol

Dogs were fasted for at least 12 hours before FDG PET/CT scanning, but had free access to water. After intravenous catheter placement, the dogs were pre- medicated intravenously (IV) with butorphanol (0.2mg/kg) (n = 10) or acepromazine (0.2mg/kg) (n = 1). Both anesthetic agents are known not to influence the blood glucose level negatively [26,27]. The FDG dose was calculated according to the dosing scheme published by the pediatric task group, European Society of Nuclear Medicine [28], (dose rate mean: 225.47 ± 80.02 MBq; 6.09 ± 2.16 Ci) (Table 1). Anesthesia was induced with propofol (4–6 mg/kg) IV; endotracheal intubation was performed, and anesthesia was maintained with constant rate infusion of propofol (4–6 mg/kg/h) IV. All dogs were ventilated with 100% oxygen and were infused with lactated Ringer’s solution (10 ml/kg/h) IV. During anesthesia, heart rate, respiratory rate, peripheral O2 saturation, end-tidal CO2-concentration, peak respiration pressure and minute ventilation volume were continuously monitored as well as the animals warmed with a heatingpad.

**Table 1:** Patient Characteristics.

Patient	Personal description	Gender	Weight (kg)	Histologic/Cytologic Diagnosis	Pre-treatment	Time of PET/CT
1	Flat Coated Retriever	mn	37.6	Histiocytic sarcoma at phalanx IV of the left hind leg	Surgical resection of the primary tumor and CCNU 50mg/kg every 3 week	First scan: three month after surgery
2	Flat Coated Retriever	fs	26.2	Mammary adenocarcinoma	No pre-treatment	After continuous progression of the mass
3	Golden Retriever	mn	35.5	Laryngeal Lymphoma	Surgical resection of the primary tumor	One month after surgery
4	Mixed breed	mn	6.45	Primary lung tumor	No pre-treatment	After continuous progression of the mass
5	Mixed breed	f	7.8	Mammary adenocarcinoma	No pre-treatment	After continuous progression of the mass
6	Mixed breed	mn	39	Sertoli cell tumor	Surgical resection of the primary tumor	One month after surgery
7	Standard Schnauzer	m	18.5	Gastrointestinal stromal tumor (GIST)	Surgical resection of the primary tumor and Palladia	Six month after surgery and five month after start of therapy
1 (Restaging)	Flat Coated Retriever	mn	37	Restaging patient 1; histiocytic Sarcoma	See above at patient 1	Second Scan: 5 months after the first scan
8	Mixed breed	fs	27.2	Nasal Lymphoma	Surgical resection of the primary tumor	One month after surgery
9	Mixed breed	f	16.5	Mammary adenocarcinoma	No pre-treatment	After continuous progression of the mass
10	Beauceron	f	31.5	Mammary adenocarcinoma	No pre-treatment	After continuous progression of the mass

Personal description and treatment of every patient before PET/CT

Chlorethyl-Cyclohexyl-Nitroso-Urea (CCNU)/ Lomustin.

### PET/CT Acquisition

After induction of anesthesia, dogs were positioned in the scanner in dorsal or ventral recumbency depending on the tumor localization.

FDG PET/CT scans were acquired on a Biograph Sensation 64 PET/CT scanner (Siemens Healthcare). All patients obtained a dedicated CT scan (120 kV, 180 mAs care dose, 0.5 s per rotation, 5-mm slice thickness, portal venous phase 80 s after the injection of 1.5 ml/kg of intravenous iodine containing contrast agent [Imeron 300 mg/ml]). The diagnostic CT scan was acquired for attenuation correction in resting expiratory position. FDG was administered intravenously to all patients 83.7 ± 12.11 minutes before the PET/CT scan. Emission time was 2 min per bed position, with 5–9 bed positions per patient (head to tail). These combined instruments make it possible to perform a PET and CT examination in one and the same scan within one instrument. The two examinations are made one directly after the other. The images are combined with the help of integrated software and a merged image is produced. This technique enables the exposure time to be considerably shortened and the image quality appreciably improved by only having to position the patients once, adding value to the data evaluation process.

### Image Analysis

The entire rating procedure was performed on a dedicated workstation using software (Syngo MMWP [workstation] and Syngo TrueD [software]; Siemens Medical Solutions).

CT und PET data were evaluated independently of one another by two specialists. On the other hand, the combined PET/CT was evaluated with the two of them together and the results of the individual CT and PET data combined with the clinical preliminary report.

A 5-point scale was used for the interpretation of the FDG-PET scans, CT scans and combined FDG PET/CT scans according to the following criteria: 1 = definitely benign; 2 = probably benign; 3 = indeterminate; 4 = probably malignant; 5 = definitely malignant. Scores of 1 and 2 were considered as benign lesions, and scores of 4 and 5 were considered as malignant. With a score of 3, a statement regarding the malignancy of the lesions was not possible. Therefore, the CT scans were interpreted subjectively regarding morphology, contrast enhancement, distribution of contrast agent. For interpretation of the PET data, FDG uptake was assessed according to the standardized uptake value (SUV).

The SUV is a semi-quantitative method of determining the uptake of a tracer in a specific tissue by using the following calculation:

$$SUV\;=\;\frac{Image\;activity\;concentration\;\left(\frac{Bq}{g}\right)\;\times \;body\;weight\;\left(g\right)}{Injected\;activity\;(Bq)}$$

For further analysis, the maximum SUV was used.

These SUV values as well as the history of each patient were considered for the evaluation of the imaged structures in combined PET/CT and assigning values within the 5-point scale. The aim of this evaluation method was to find out whether the additional PET images provide an advantage in the diagnosis/assessment of anatomic structures.

For the analysis of the PET/CT results of all patients, a total of 89 individual regions were evaluated using a 5-point scale and included abdominal and thoracic organs, regional and peripheral lymph nodes, and salivary glands. The conclusion from this evaluation method was that the additional PET data appreciably simplified the subjective assessment of anatomic structures compared to CT by itself, especially with the abundance of data from a full body scan. Follow-up studies are necessary in order to prove this statistically.

A Region of Interest (ROI) is a subset of an image in which the data points are sampled for analysis. In this study, the ROI was defined as the primary tumor, its draining lymph nodes, any lesions suggestive of metastatic disease, the liver, spleen, thymus, salivary gland, renal cortex, and left ventricular heart muscle. The ROI was placed at a representative, central position of the relevant organ, filling the anatomically predefined shape of the organ as well as possible and the SUV value (min, mean and max) were compiled by drawing the ROI within five successive axial, dorsal, or sagittal slices. In the same way, the ROI was placed on the primary tumors if still present and other conspicuous structures. With the lymph nodes on the other hand, because of the partly smaller size, the ROI was placed centrally using an ellipse and in the same way the min, mean and max values from five successive sections were used for further assessment. Brain, renal pelvis, lower urinary tract, blood vessels, and gall bladder were not included in the evaluation [15].

**Fig 1:**
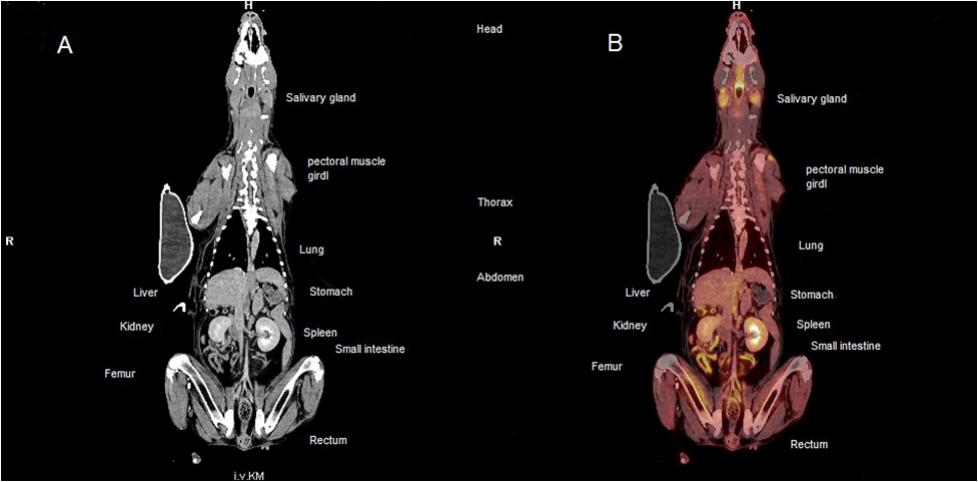
Overview of thoracic and abdominal organs in a whole body 18F F-fluoro-2-deoxy-D- glucose (18F-FDG) positron emission tomography/computer tomography (PET/CT) in a patient with multicentric lymphoma. (A) Coronal contrast enhanced CT; (B) Coronal 18F-FDG PET/CT. Overview of the thoracic and abdominal organs of a dog with a multicentric lymphoma. The mean SUVs of these organs are defined in Table 4. The labeled organs are normal with physiological FDG-uptake (see Table 4).

Whole body images (Fig 1) acquired one hour after FDG injection were reviewed.

## Results

Patient characteristics and any treatment given prior to the FDG PET/CT are shown in Table 1. The treatment protocol was changed significantly in seven out of ten dogs due to the results of FDG PET/CT. The resulting therapy after FDG PET/CT and the clinical follow up six months later is shown in Table 2. Presumed areas of malignancy and other structures with increased FDG uptake were found and involved in further data analysis (Table 3). Table 3 shows the regions with increased FDG uptake and the cytologic or histologic findings in these regions. In addition, in every patient, the SUV values of selected organs, the main target areas of metastases (lung, liver and spleen), and the lymph nodes as well as the primary tumors were measured individually (Table 4). These SUVs are in accordance with previously published FDG PET/CT data in dogs [9,13,15]. Furthermore the SUV in the primary tumors was increased compared to the physiologic thoracic and abdominal organs (Table 4). As a rule, malignant tumors exhibit an increased SUV value, a cutoff value of >2.5 being described [29]. However, an increased SUV does not represent a diagnosis and certainly does not make any further elucidation unnecessary. An increased SUV value is an indication of a malignant tumor, but an inflammatory process must always be taken into consideration as a differential diagnosis [30].

**Table 2:** Findings of PET/CT, resulting therapeutic decisions and clinical follow-up.

Patient	Histologic/Cytologic Diagnosis	FDG MBq	Results of PET/CT	Therapy after PET/CT	Follow up six month after PET/CT
1	Histiocytic sarcoma	351	No evidence of metastases/ no increased uptake in regional lymph nodes	CCNU 50mg/kg every third week	Well as far as the end of august 2013; at this point of time relapse of histiocytic sarcoma was cytologically diagnosed dorsal on the left metatarsal bones; curative RTH was performed; after that CCNU 50mg/m² was started until now; Euthanasia nearly one year after diagnose
2	Mammary adenocarcinoma	208	No evidence of metastases/ no increased uptake in regional lymph nodes s	Surgical resection of the primary tumor	She is doing well
3	Laryngeal Lymphoma	293	Increased FDG uptake in regional and peripheral lymph nodes	No therapy although chemotherapy was approved	Dead six weeks after PET/CT scan
4	Primary lung tumor	104.8	Increased uptake in the primary tumor and in another lung lesion	No therapy, because of the result of FDG PET/CT; poor prognosis	He is doing well; Restaging with the aid of X-rays ten months after the PET/CT scan; the primary lung tumor has distinctly grown; furthermore the metastasis is now detectable in the chest X-rays
5	Mammary adenocarcinoma	96.4	No indication of metastases/ no increased uptake in regional lymph nodes	Surgical resection of the primary tumor	She is doing well
6	Sertoli cell tumor	301	No indication of metastases/ no increased uptake in regional lymph nodes	No therapy, because of the result of FDG PET/CT	He is doing well
7	Gastrointestinal stromal tumor (GIST)	169	No indication of metastases/ no increased uptake in regional lymph nodes	No therapy with Toceranib, because of the result of FDG PET/CT	He is doing well
1 (Restaging)	Restaging Patient 1; histiocytic sarcoma	300	No indication of metastases/ no increased uptake in regional lymph nodes	No therapy with CCNU, because no indication of metastases	See above at patient 1
8	Nasal Lymphoma	226	Increased FDG uptake in regional and peripheral lymph nodes	curative RTH	Multicentric Lymphoma with enlarged peripheral lymphnodes three months after PET/CT scan; Prednisolone 2mg/kg daily was started; Euthanasia seven months after PET/CT
9	Mammary adenocarcinoma	171	No indication of metastases/ no increased uptake in regional lymph nodes	Surgical resection of the primary tumor	She is doing well
10	Mammary adenocarcinoma	260	Increased uptake in the primary tumor Increased uptake in regional and peripheral lymph nodes as well as in other organs	No therapy because of the result of FDG PET/CT; poor prognosis	Euthanasia five months after PET/CT

Results of PET/CT; in every patient the SUVs of the local and regional lymph nodes as well as the main target areas of metastases were measured for accommodating the prognosis and following therapy individually. Abnormal findings in CT as well as increased FDG uptake were classified with the help of the 5-point scale.

**Table 3:** Mean and Max SUVs.

Localization of increased FDG uptake	Histologic/Cytologic Diagnosis	Suspected	Patient	SUV mean	SUV max	SD
Right tooth root		Inflammation	2	6.09	9.10	1.22
Mass on the left mamma (primary tumor)	Mammary adenocarcinoma of mamma		2	1.98	2.97	0.44
Masses on the dorsal neck		Lymphoma	3	4.87 5.76	6.95 8.44	1.07 1.03
Intramuscular		Lymphoma	3	5.82 3.04 3.81 6.97	7.45 6.11 5.44 8.74	1.10 1.25 0.87 1.13
Lnn. Mandibularis		Lymphoma	3	2.03 2.06	2.53 2.62	0.32 0.29
Lnn. Retropharyngealis		Lymphoma	3	2.37 2.43	3.01 3.84	0.46 0.42
Mass in the lung (primary tumor)	Carcinoma	Bronchial adenocarcinoma	4	6.28	9.40	1.28
Mass in the lung		Metastasis of bronchial adenocarcinoma	4	0.76	1.02	0.16
Mass on the left mamma (primary tumor)	Mammary adenocarcinoma of mamma		5	2.75	4.23	0.51
Salivary glands		Hypermetabolic salivary gland	6	12.40 12.38	13.57 13.42	0.79 0.75
Cutis	Telogen effluvium		6	5.77 5.40	7.38 6.62	1.10 0.90
Perianal zone		Inflammation	6	10.45	16.07	2.01
Mass near left Ln. inguinalis		Inflammation post OP	6	2.63	3.80	0.56
Lnn. inguinalis		Reactive lymph nodes	6	1.16 1.97	1.68 2.57	0.20 0.35
Mass in the Nose (primary tumor)	Lymphoma (B-cell)		8	9.11	12.86	2.06
Mediastinal masses		Lymphoma (B-cell)	8	2.81 9.54 4.96	4.01 13.59 7.18	0.54 1.97 1.17
Lnn. retropharyngealis		Lymphoma (B-cell)	8	7.37 2.47	10.86 3.40	1.53 0.47
Mass on the right mamma	Carcinoma	Mammary adenocarcinoma of mamma	10	6.92	10.01	1.06
Mass on the left mamma	Carcinoma	Mammary adenocarcinoma of mamma	10	3.62	5.20	0.80
Mass in the spleen		Metastasis of mammary carcinoma	10	2.91	3.58	0.31
Mass near right Ln. axillaris	Carcinoma	Metastasis of mammary carcinoma	10	4.37	6.78	0.82
Mass near anus	Basalioma		10	2.78	3.08	0.18
Lnn. axillaris	Carcinoma	Metastasis of mammary carcinoma	10	3.21 2.12	5.16 3.23	0.51 0.39
Lnn. retropharyngealis		Metastasis of mammary carcinoma	10	1.62 1.95	2.96 2.22	0.30 0.30

Abnormal findings with increased uptake in PET/CT with their cytologic or histologic or differential diagnosis in individual patients.

**Table 4:** SUV max values in organs.

Organ	N	Minimum	Maximum	Mean	SD
Liver	10	1.71	3.39	2.6480	.60716
Spleen	10	1.33	2.51	1.9210	.43370
Myocardium	10	1.63	5.78	3.4790	1.35948
Cortex of kidney	10	2.49	5.41	3.8730	1.05205
Salivary gland	9	2.18	6.66	4.6572	1.44825
Lymph nodes	10	1.36	4.70	2.0303	.97479
Primary tumor	6	2.97	12.86	7.7367	3.73684
Solid metastases	4	1.02	8.62	5.656	3.275
Lymph node metastases	3	3.0	4.54	3.9	.77485

Primary tumor measured in patient numbers 2,4,5,8,9,10.

Solid and lymph node (suspected) metastases are defined separately in table.

Table 4 is intended to show that in our study, the primary tumors exhibit an increased SUV value compared to physiologic tissue and so can be used as a criterion of malignancy.

In 9 of 10 dogs in this study that underwent FDG PET/CT while a primary tumor was present, a distinctly increased FDG uptake within the primary tumor could be measured.

The only tumor lacking increased FDG uptake was the grade I cutaneous mast cell tumor in one of the dogs presented with an additional mammary carcinoma. This patient first presented with a mammary carcinoma. In the clinical pre-examination for the PET/CT examination, a mass with the diameter of approximately 0.5 cm could be clearly palpated which however did not show increased FDG uptake. Nevertheless, the mass was investigated cytologically before the surgical removal of the mammary tumor and was found to be a mast cell tumor. In the subsequent operation, both the mammary tumor and the mast cell tumor were surgically removed.

There were a few unexpected findings in one of the dogs. The patient with the previously resected sertoli cell tumor showed markedly increased FDG uptake in both salivary glands. The same dog showed a diffusely increased cutaneous FDG uptake despite the fact that at the time the FDG PET/CT was performed, the dog did not show any obvious skin changes on physical examination. Histopathologic examination of the skin revealed a telogene effluvium. Telogen effluvium is a scalp disorder characterized by the thinning or shedding of hair resulting from the early entry of hair in the telogen phase (the resting phase of the hair follicle). Emotional or physiological stress may result in an alteration of the normal hair cycle and cause the disorder. Telogen hair follicles in the dermis can be associated with an inflammation [31]. However, this result was not to be connected with the sertoli cell tumor.

No complications such as an anesthesiological incident or an anaphylactic reaction occurred during FDG PET/CT and all dogs could be discharged three hours after the examination according to the radiation protection ordinance (< 14kBq).

## Discussion

The aim of this pilot study was to evaluate the feasibility of FDG PET/CT in dogs as well as the efficacy of whole body FDG PET/CT for staging in a case series of 10 dogs with various tumor types with a perspective for further consecutive comparative studies. Furthermore, drawing interest in dogs as a well- suited animal model for comparative oncology was another aim of this study.

Due to a lack of published data for FDG PET/CT imaging in dogs with sertoli cell tumor, GIST or Basalioma as well as in hypermetabolic salivary gland no direct comparison with other studies could be made. For this reason, results deriving from human medicine regarding the detectability of the mentioned tumors as well as others in FDG PET/CT had to be used.

Only the patient with the sertoli cell tumor showed markedly increased FDG uptake in both salivary glands. An association of these findings with the sertoli cell tumor was considered very unlikely. Furthermore a human study concluded that a differentiation between benign and malignant salivary gland tumors is not possible by FDG PET/CT based on an increased FDG uptake [32]. Therefore, these findings were not included in further treatment decisions for the dog.

Up to now an increased FDG uptake has been described only in grade II and grade III canine mast cell tumors [9]. In this study the only tumor lacking increased FDG uptake was a grade I cutaneous mast cell tumor. Grade I mast cell tumors have not been evaluated by FDG PET/CT beforehand, therefore it cannot be excluded that the metabolic activity of grade I tumors may be too low to cause an increased FDG uptake. It is also possible that the grade I mast cell tumor was too small to be detectable by CT or PET.

**Fig 2:**
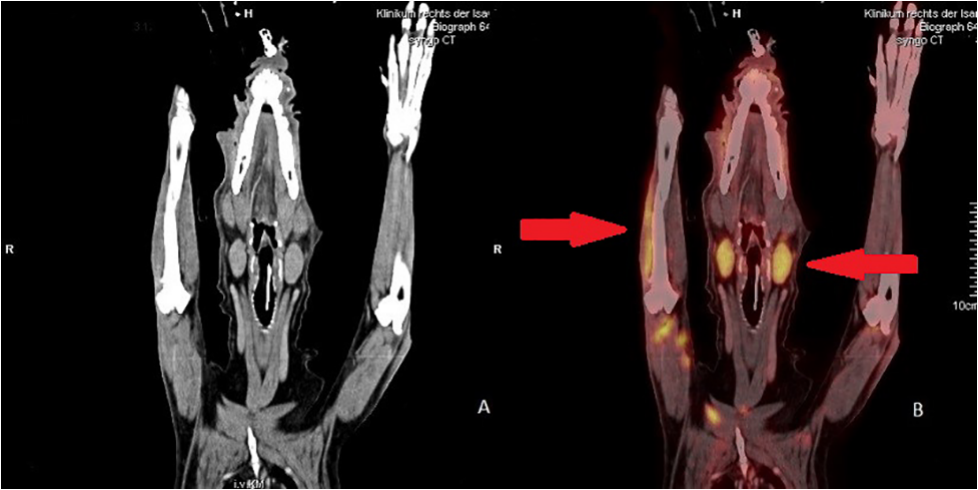
18F-FDG uptake in a patient with multicentric lymphoma. (A) Coronal contrast enhanced computer tomography (CT); (B) Coronal 18F-fluoro-2-deoxy-D- glucose (18F-FDG positron emission tomography (PET)/CT) Increased FDG uptake in both retropharyngales lymph nodes (standardized uptake value (SUV) mean 2.37 and 2.43 ± 2.4) as well as intramuscular increased FDG-uptake (SUV mean 3.04 ± 1.25).

**Fig 3:**
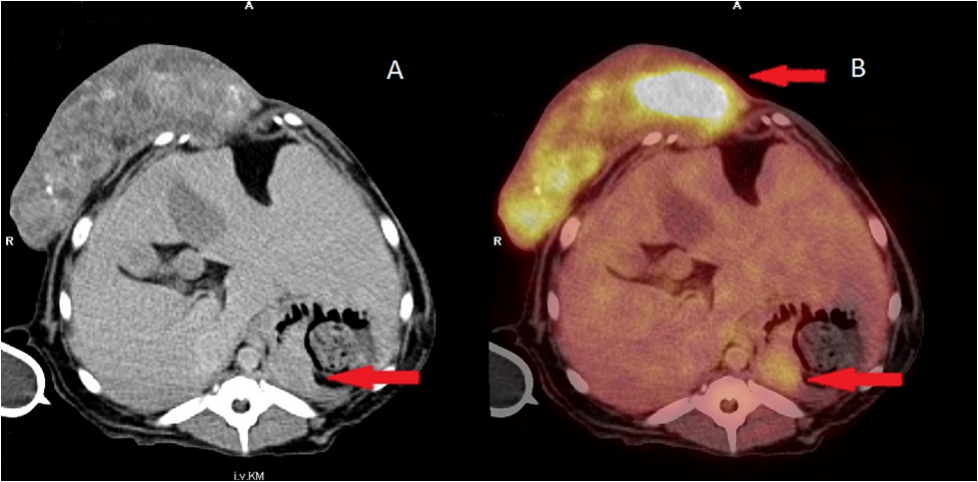
18F F-fluoro-2-deoxy-D-glucose (18F-FDG) uptake in a patient with mammary carcinoma and multiple metastases. (A) Axial contrast enhanced computer tomography (CT); (B) Axial 18F-FDG positron emission tomography (PET)/CT Increased FDG uptake in the primary tumor (SUV mean 6.92 ± 1.06), histologically a mammary carcinoma on the mammary gland; hepatomegaly; mass within the spleen with increased 18F- FDG uptake (SUV mean 2.91 ± 0.31).

In the present study, FDG PET/CT was a particularly promising imaging method in patients with lymphoma (n = 2) (Fig 2), mammary carcinoma (n = 4) (Fig 3), and primary lung tumor (n = 1). In these patients the results of FDG PET/CT had a major impact on treatment recommendation. The two dogs with lymphoma were initially considered to suffer from a locally confined tumor. In both patients a systemic involvement could be demonstrated by FDG PET/CT. Systemic chemotherapy might have resulted in improved survival times in these patients but it was declined by the owners. In human medicine, FDG PET/CT is already widely used as a non-invasive method for staging and control of response to treatment in patients with Non-Hodgkin’s lymphoma [33–36]. In veterinary patients, this method proved to be highly sensitive [9,11,19]. In this study, PET/CT resulted in a significant change in the prognosis and in therapeutic recommendations for the lymphoma patients and is expected to be particularly beneficial in these patients to exclude or to detect systemic disease. For further studies it would be interesting to use 3`-deoxy-3`-[18F]fluoro-thymidine (FLT) tracer instead of FDG as it showed a higher sensitivity in detecting lymphoma in different species [19,37,38].

**Fig 4:**
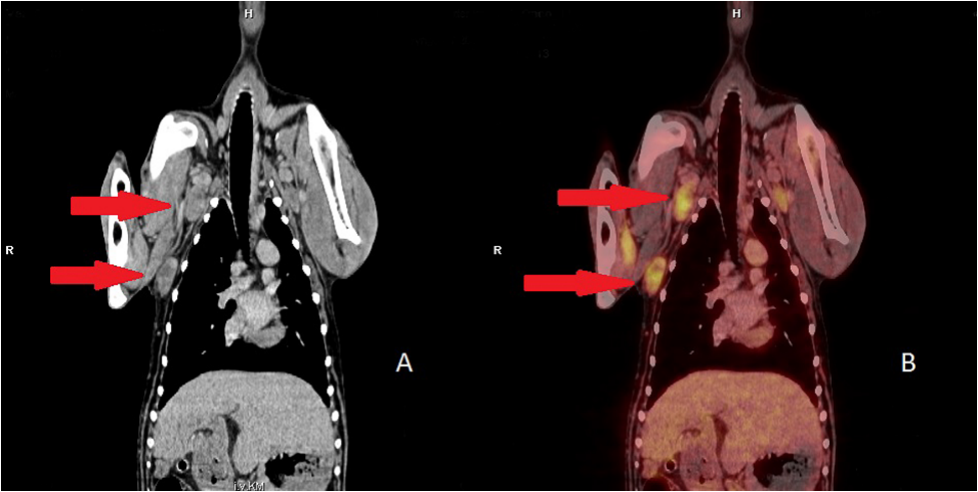
18F F-fluoro-2-deoxy-D-glucose (18F-FDG) uptake in a patient with mammary carcinoma and multiple metastases. (A) Coronal contrast enhanced computer tomography (CT); (B) Coronal 18F-FDG positron emission tomography (PET)/CT Increased FDG uptake in both lnn. axillares (SUV mean 2.67 ± 0.77) as well as in a subcutaneous mass (the more caudally located structure) (SUV mean 4.37 ± 0.82).

In dogs with mammary carcinoma, FDG PET/CT was very helpful for evaluation of regional lymph nodes in order to confirm or exclude metastatic disease (Fig 4). The exact evaluation of lymph nodes for metastases before surgery is beneficial for accurate planning of a minimally invasive surgical procedure. Combined PET/CT has proven to provide considerably more information on whether lymph node metastases are present or absent compared to CT imaging alone [39,40]. The results of our study are consistent with this statement. We wanted to show with our study that the analysis of CT data can be simplified greatly with the aid of PET. Tracheobronchial lymph nodes and abdominal abdominal lymph nodes, in particular, are visualized more clearly by combined PET/CT than CT alone. This facilitates the evaluation of possible pathological findings at these locations. Abnormalities of internal organs are also better visualized with PET, making it easier to evaluate changes here as well. (Fig 3).

In addition, FDG and FLT PET/CT were described in a dog with a primary bronchial adenocarcinoma [8]. In that case report, the dog was evaluated with PET/CT before and after treatment. Both FDG and FLT PET/CT tracers were found to be effective in differentiating tumor tissue from physiologic or reactive tissue. In the present study, FDG PET/CT was also used in a dog with a primary pulmonary tumor. On PET/CT, pulmonary metastatic disease was found, whereas no increased FDG uptake could be demonstrated within the locally draining lymph nodes. In human as well as in veterinary medicine the staging, especially the mediastinal nodal staging, is the most important prognostic factor concerning the median survival time in patients with primary pulmonary carcinoma [41–43]. This finding led to the decision against surgical treatment (e.g. lung lobectomy) despite the lack of mediastinal lymph node metastases. The relatively low FDG uptake in the metastatic lung lesion (SUV max 1.02 ± 0.16) compared to the primary tumor (SUV max 9.40 ± 1.28) is characteristic for human lung cancer [44]. Even though PET/CT is more accurate in detecting metastases compared to CT alone [45], false- positive findings in PET/CT have to be considered [46]. In this study, the lung lesion (left pulmonary lobe, pars cranialis, dorsally, marginally, about 0.6mm diameter) could be confirmed by X-rays during restaging six months later which proved the PET/CT findings.

In dogs with histiocytic sarcoma (n = 1), sertoli cell tumor (n = 1) and GIST (n = 1), FDG PET/CT showed no indications of metastases although the histologic findings as well as the biologic behavior of the tumor predicted a poor prognosis. Therefore, the follow up treatment was not changed. In human patients with GIST, FDG PET/CT is already widely used for non-invasive staging. A correlation between the degree of malignancy and the intensity of FDG uptake has been demonstrated, so FDG PET/CT provides important prognostic information in this tumor type. FDG PET/CT has also proven to be helpful for evaluation of the response to treatment and early recognition of tumor recurrence in patients with GIST [47–49]. However, FDG uptake in human GISTs was reported to vary widely [50]. Negative, low, or intensive FDG uptake can occur. In the present study, FDG PET/CT was performed in the patient after surgical resection of a GIST. Therefore, a comparison with pre-treatment values was not possible, nor was evaluation of the primary tumor possible.

In human medicine, there are only a small number of reports on FDG PET/CT in patients with a Sertoli cell tumor [51] or histiocytic sarcoma. Increased uptake was reported to be measured in the mass themselves as well as in metastatic lymph nodes [52]. In addition there is one case report with a FDG PET in a canine intracranial histiocytic sarcoma [16]. These case reports indicate that FDG PET/CT might be valuable for early detection and staging of testicular tumors and histiocytic sarcoma in human as well as in veterinary medicine.

These cases should illustrate the possible consequences of staging by FDG PET/CT in canine patients as well as the comparable and transferable results in human and veterinarian patients with spontaneously occurring cancer.

Limitations of the study are the small number of patients that precluded statistical evaluation of the results and the heterogeneous patient population including various tumor types and dogs with and without prior treatment of their tumors which precluded a direct comparison with other imaging methods for staging. Some dogs underwent surgical resection of their primary tumor before FDG PET/CT. In these cases, FDG PET/CT evaluation of the primary tumor was not possible. In patients with incompletely resected primary tumors, the FDG uptake at the site of surgery might have been compromised by physiologic postoperative local reactions.

## Conclusion

In conclusion, the results of this pilot study indicate that FDG PET/CT in dogs with previously described malignancies can be a promising technique for the staging of dogs with specific cancers and a very useful tool for individual adaptation of treatment and prognosis. The interpretation and evaluation of the FDG PET/CT values shows similar advantages and restraints as in human patients. Thus, it appears to be plausible for the canine tumor patient to be able to serve as a model for diagnostic and therapeutic studies of human cancer in the future. However, clinical signs of the patient as well as the biologic behavior of various tumor types have to be considered when interpreting the data obtained by FDG PET/CT. This is required for correct interpretation of increased FDG uptake and for establishing a correlation with an underlying disease process. Further studies to evaluate the sensitivity and specificity of this method as well as the differentiation of more specific tracers for oncologic PET/CT in dogs are necessary. Although the imaging with FDG PET/CT is not a new method in human medicine, this study should draw interest to dogs as an animal model for further comparative studies.
